# United States College of Veterinary Surgeons

**Published:** 1900-07

**Authors:** 


### UNITED STATES COLLEGE OF VETERINARY SURGEONS.

The sixth annual commencement of this college was held in the college lecture-room, April 12, 1900. Despite the threatening weather the hall, which had been tastily decorated with bunting and flags, was comfortably filled by the friends and relatives of the graduates. The students were conspicuous, as they all wore the college colors, and the graduating class occupied a prominent place near the platform.

The exercises were opened by the Dean, C. Barnwell Robinson, with a few brief introductory remarks. Among other matters some personal reminiscences of his early experience as a veterinarian elicited close attention. He reminded the students that, though they had successfully passed the examinations and their school days had closed, yet their need for books, study, and investigation would never end. The world cared little for their diplomas; few, indeed, would ever inquire if they had one, and perhaps no one would ever read them. Their standing in the profession would depend upon the character of their work. The world will not ask, "Who are you?" but "What can you do?"

Dr. J. P. Turner, professor of zoötechnics, milk- and meat-inspection, was then introduced. His appearance upon the platform was the occasion of an outburst of applause. He at once engaged and maintained the closest attention on the part of the audience. His address was of an eminently practical nature. The great need of moral stamina on the part of the young practitioner was forcibly dwelt upon; he must elevate the profession by elevating himself. The course of the graduate who hung out his shingle at a disreputable livery stable and condemned himself to a low and ignorant class of associates was severely criticised. Instances were cited of bright, clever, and capable young men who, from lack of moral force and courage, were driven from the field of veterinary science. Humanity, moral principle, and love of the profession should be the golden rule of their professional as well as private lives.

G. A. Prevost, LL.B., professor of jurisprudence, was then introduced. In the course of his remarks some good fatherly advice was given. The early history of the school was outlined; its struggles briefly recapitulated and commented upon, and, as the graduates were feelingly admonished to walk in the straight and narrow path, thereby maintaining and upholding the honor of their Alma Mater, the diplomas were handed them. The graduates were Wm. J. Guilfoil, Auburn, N. Y.; H. K. Kauffman, Hegins, Pa.; F. B. Berger, Baltimore, Md., and Frank Feagans, Wardensville, W. Va.

"Judge" Feagans delivered the valedictory address. In his paper, which showed careful preparation and deep thought, the development of veterinary science was reviewed; the advances made in the treatment of the sick and wounded, the growth of sanitary medicine, and the evolution of humanitarian ideas were noted. The great impetus given to the study and diagnosis of disease by the wonderful achievements of bacteriology were fittingly dwelt upon.

In taking leave of his class associates and the Faculty, Dr. Feagans touchingly alluded to the many courtesies extended by the Faculty. "Ere yet again rosy-fingered Aurora, goddess of morn, has gilded the lofty domes of this beautiful city, many of the faces before me will be gone. Succeeding years may witness many assemblages around this platform; many speeches will go echoing down the halls of this historic structure, but no assemblage, however brilliant, no speech, however witty, can efface from memory the valuable lessons we have learned or the noble impulses which have inspired us while here."

In responding to the valedictory address, Dr. H. W. Acheson, professor of obstetrics and cattle pathology, soon had the audience roaring with laughter at his unconscious (?) wit and brilliant repartee. His remarks were replete with valuable suggestions, and the fear of all who heard him was lest he should make an end of speaking.

At the conclusion of Dr. Acheson's remarks each graduate was presented, on behalf of the Faculty, with a large bouquet of rare and fragrant flowers from the conservatory of one of Washington's distinguished florists. Mention should also be made of the picture, grouping the entire school and Faculty, presented the Dean by the students.

The degree conferred upon the graduates is that of Doctor of Veterinary Science and Membership in the United States College of Veterinary Surgeons.

(Reported by Jas. R. Covert, an undergraduate.)

---

There is too much effort on the part of schools to give an extended education in the branches of practical medicine and surgery to minds that are imperfectly prepared to accept the same through lack of essential fundamental branches. Veterinary colleges should look to their own standing and reputations in this direction.

